# Supplementary material for: Climate co-benefits of tiger conservation
Source: Nat Ecol Evol. 2023 May 25;7(7):1104–13. doi: 10.1038/s41559-023-02069-x (PMC10333118; doi:10.1038/s41559-023-02069-x)
Supplement: Supplementary file 1 — Supplementary Figs. 1–6 and Tables 1–4. [file 41559_2023_2069_MOESM1_ESM.pdf]

nature ecology & evolution

Article

https://doi.org/10.1038/s41559-023-02069-x

Climate co-benefits of tiger conservation

In the format provided by the authors and unedited

Supplementary Figures

Supplementary Fig. 1: A conceptual outline of the methodology used.

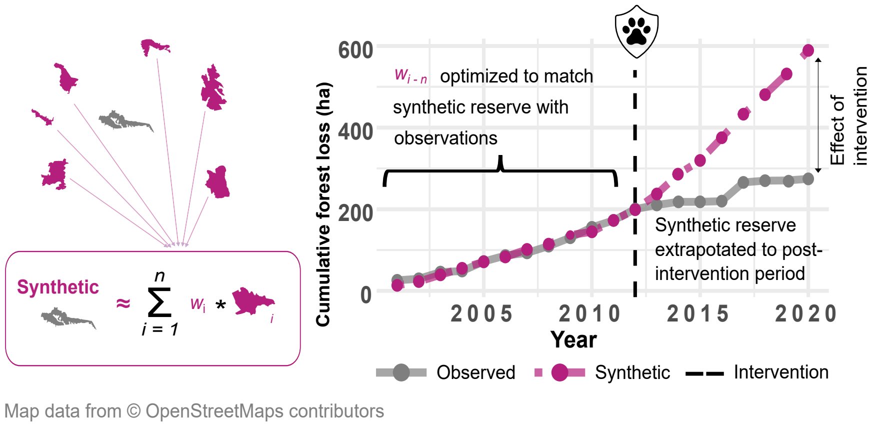

(Left) The synthetic counterfactual reserves represent what would have happened had the intervention i.e. the tiger conservation policy had not taken place in the 'treated' reserves shown in grey. These can be thought of as a weighted sum of the untreated 'donor' in pink reserves that did not undergo the policy. These weights are adjusted to best model the trajectory of forest loss in the treated reserve before the intervention took place. (Right) The dotted pink line represents the synthetic control which matches the observations represented by the dotted grey line in the pre - intervention period before. The time of the intervention is shown with the dashed black line. This synthetic model is then extrapolated into the post -intervention period, and the difference between the synthetic control and observations is the effect on forest loss that can be attributed to the policy intervention.

Supplementary Fig. 2: Correlation plot for assessing collinearity in reserve-level covariates.

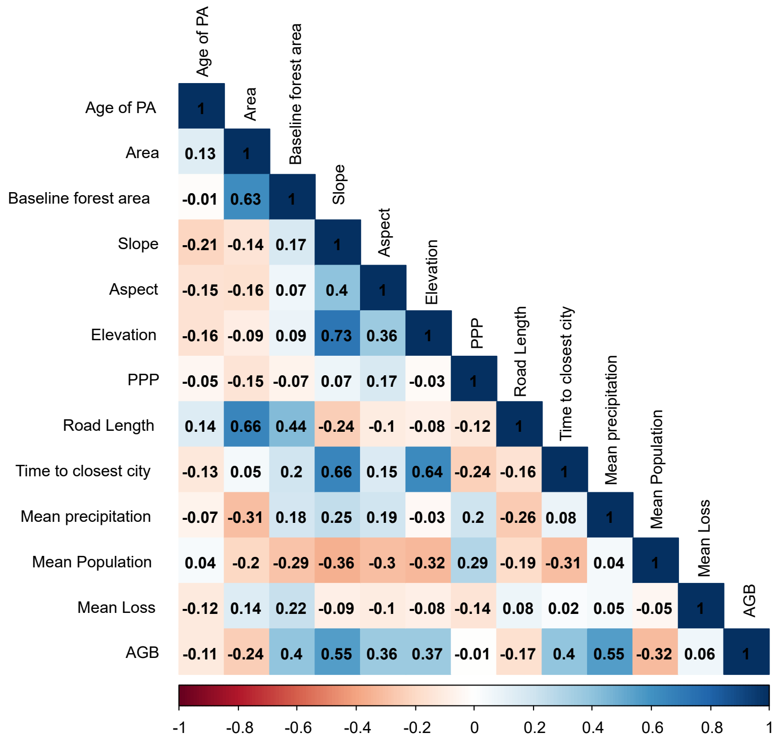

Collinearity between predictor variables was assessed by determining their pairwise Pearson correlation coefficients (reported in the correlation matrix in the figure above). We did not exclude any predictor variable from the analysis because the absolute values for all correlations were <0.8.

Supplementary Fig. 3: Visualizing structural differences in the drivers of forest loss between treatment and donor reserves.

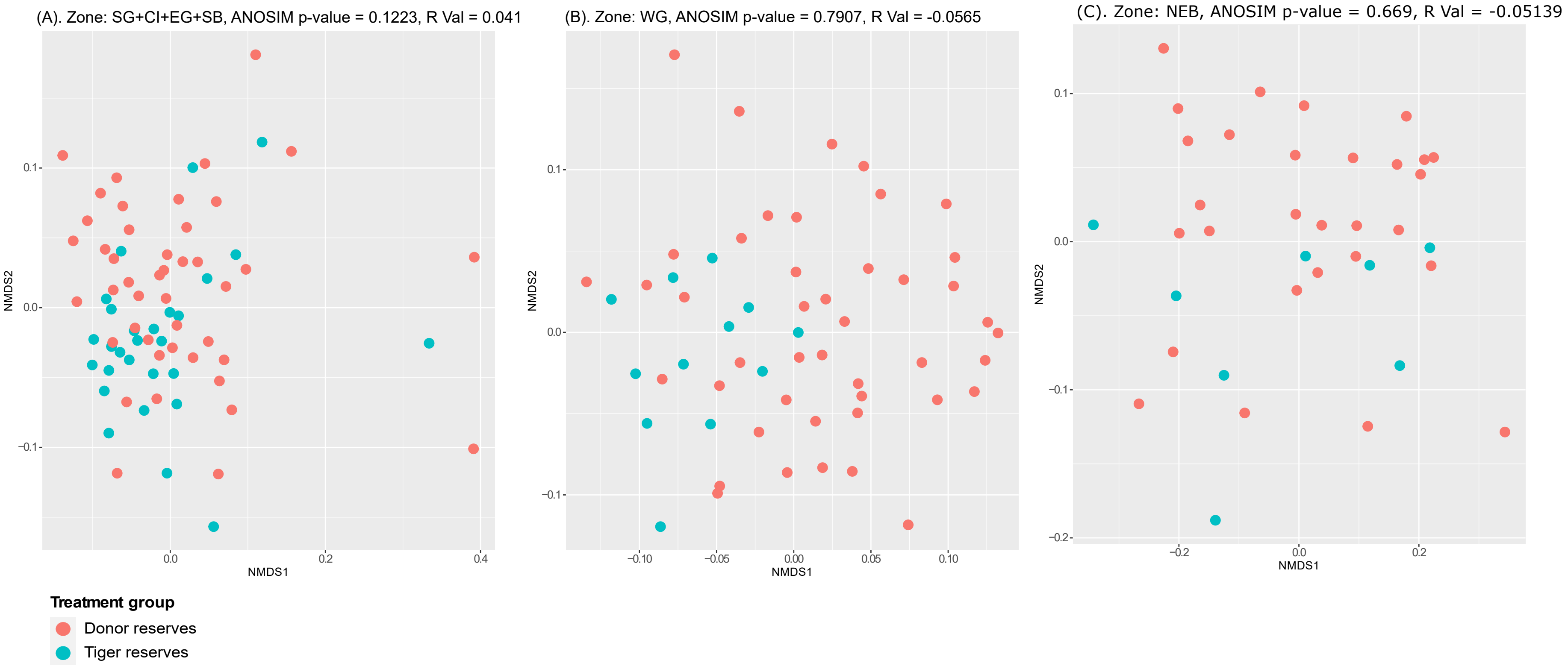

To examine if there are structural differences between the treatment and donor reserves between 2000 and 2020, we utilized a dimensionality reduction approach implemented using Non-metric Multidimensional Scaling (NMDS). These NMDS plots are based on reserve-level covariates which include age of protected area, reserve size, area under forest cover, mean precipitation, mean human population density, road length within reserve boundaries, slope, aspect, elevation, spatial purchasing power parity and mean above-ground biomass in the baseline year. To ensure robust significance testing, more than 20 donor reserves were required in each geographical grouping. Therefore, contiguous tiger conservation landscapes[56] were combined to produce the following groups: (A) Shivalik-Gangetic, Central India, Eastern Ghats and Sunderbans regions, (B) Western Ghats and (C) North-East Hills and Brahmaputra region. The orange dots represent untreated 'donor' reserves while the blue dots represent Tiger Reserves that underwent the enhanced tiger conservation policy. In all three of the geographical zones analysed, visually the treatment reserves are embedded within the donor reserve clusters suggesting no substantial difference in structural drivers of forest loss between the two groups. Additionally, Analysis of Similarity or ANOSIM was used to assess the similarity between treatment and donor clusters. The variation between donor and treatment pools was found to be insignificant (p-values for Zones (A), (B) and (C) were 0.1223, 0.7907 and 0.6690 respectively suggesting no statistical difference).

Supplementary Fig. 4: Placebo tests to assess the statistical significance of synthetic control models.

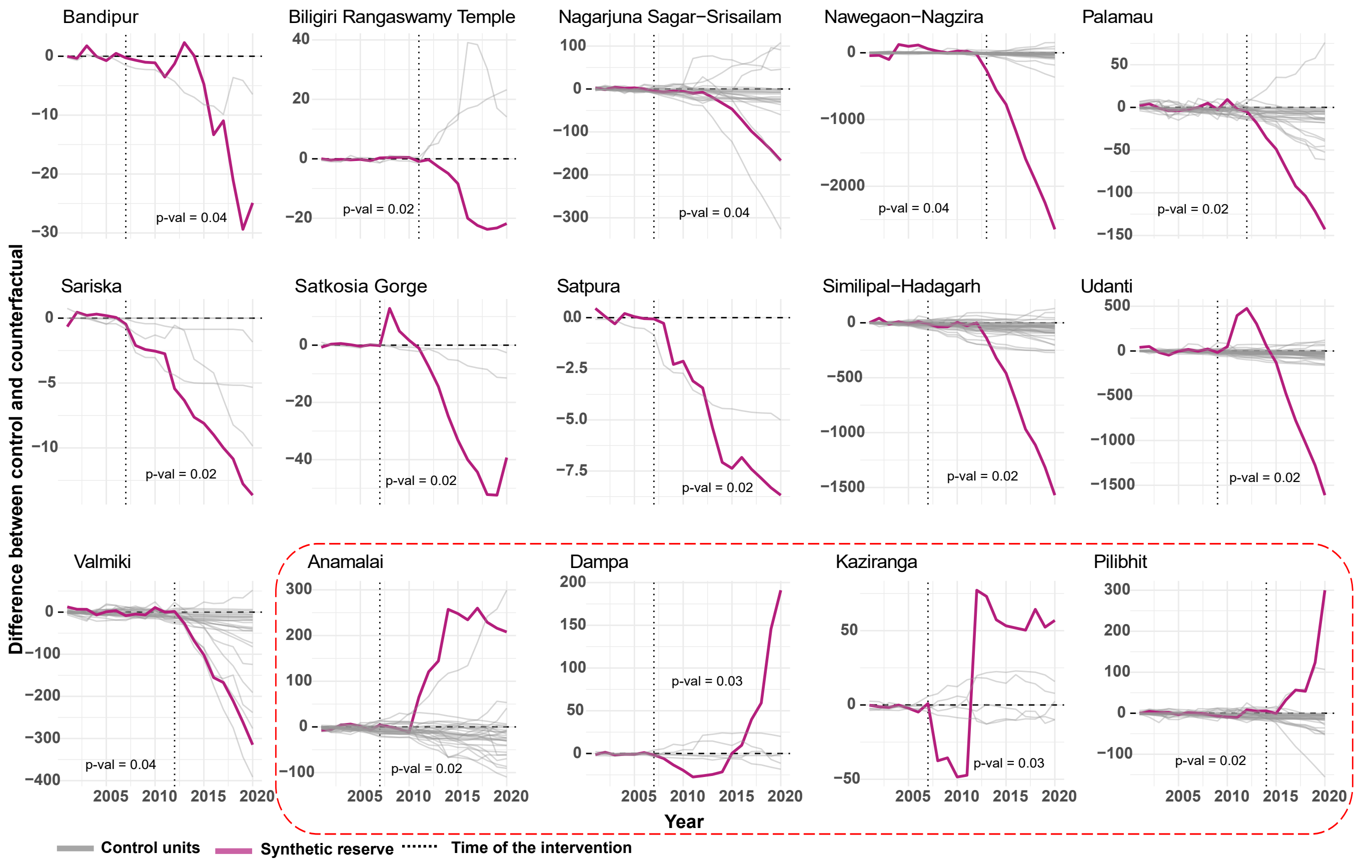

Placebo tests were used for significance testing of synthetic controls models. A placebo unit represents the synthetic model created using one of the donor units. In this case, a donor unit is treated as the treated unit and the actual treated unit is added to the donor pool. For the intervention to have an effect i.e. to reject the null hypothesis that no effect of the intervention on the cumulative forest loss, the differences in the performance of the donor units and the treated unit should be statistically different. To reject the null hypothesis, the mean squared prediction error ratios of the pre-intervention and post-intervention period must be significantly different between the synthetic Tiger Reserve and the placebo units based on a two-sided Fisher's exact test to rule out whether the observed effect of the intervention on the outcome variable was a chance event. The vertical axis represents the difference between observed cumulative forest loss and counterfactuals for the reserves that demonstrated significant results (unadjusted $p < 0.05$). The solid pink line represents the synthetic control model while the light grey lines represent placebo tests. Placebo cases with a pre-period mean squared prediction error (mspe) exceeding five times the treated unit pre-period mspe have been pruned. The dashed red box represents placebo tests for reserves that had higher than expected deforestation. Unadjusted p-values levels from placebo testing have been reported for each synthetic counterfactual in the displayed plots.

Supplementary Fig 5. Trend lines for cumulative forest loss using a backdated intervention in 2005 to test for anticipation effects.

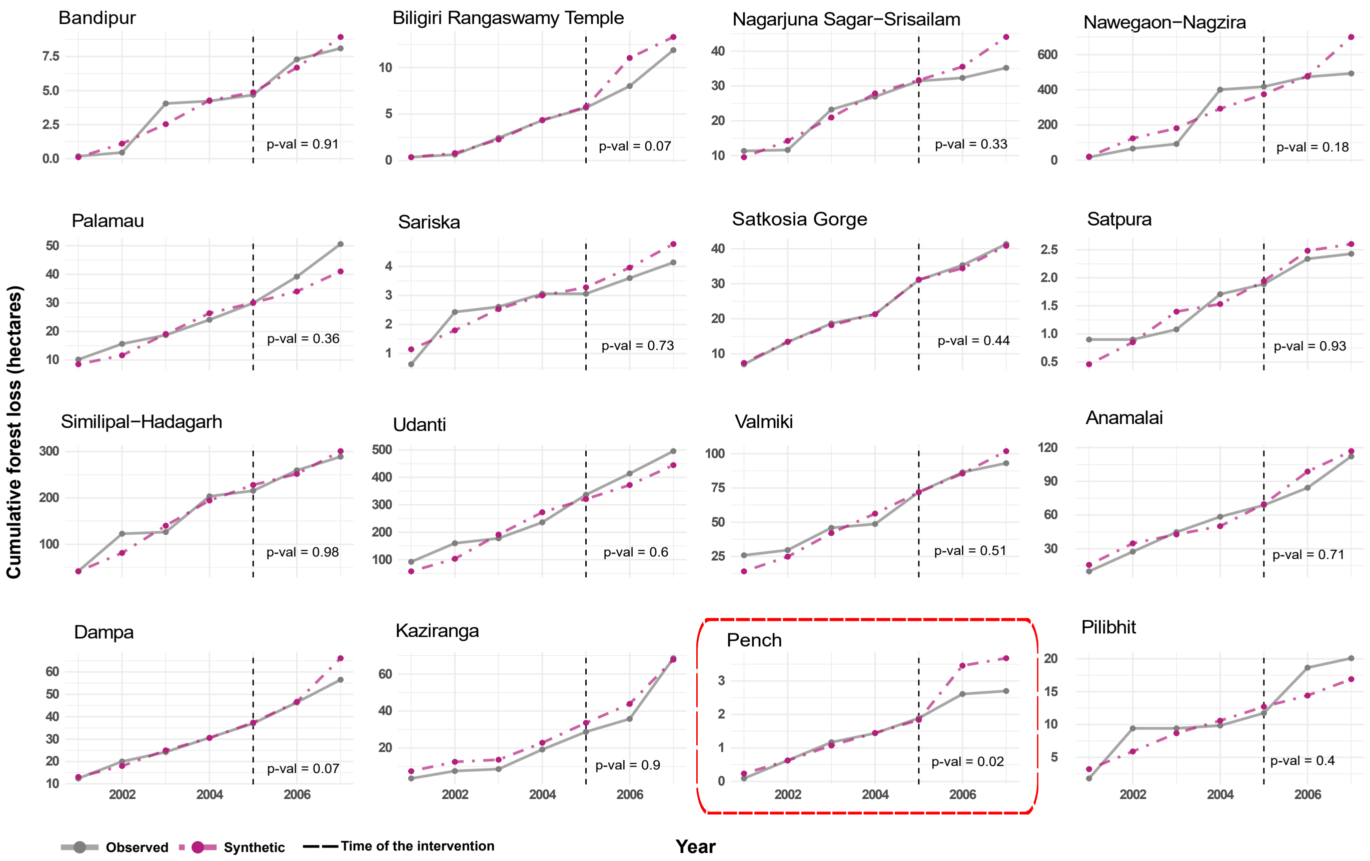

To test for anticipation effects, a backdated hypothetical intervention in 2005, which coincides with the constitution of the National Tiger Conservation Authority was implemented. Reserves that also exhibited significant effects from this pseudo-intervention were ruled out from the analyses due to the influence of potential anticipation effects. Of the 16 reserves from Supplementary Table 2 that exhibited significant effects of the enhanced conservation policy on deforestation rates (based on placebo testing using a two-sided Fisher's exact test to compare the ratios of pre-intervention and post-intervention mean squared prediction errors between placebo and treated units), only Pench Tiger Reserve exhibited significant anticipation effects (unadjusted p-value = 0.02). The trendlines for this reserve are enclosed in the dashed red box. The dotted pink line represents the cumulative forest loss for the synthetic control model while the dotted grey line represents observed deforestation in hectares. The intervention year is denoted by the dashed black line. Significance levels from placebo testing have been reported for each synthetic counterfactual in the displayed plots.

Supplementary Fig. 6: Placebo tests to assess the significance of a backdated intervention in 2005 to test for anticipation effects.

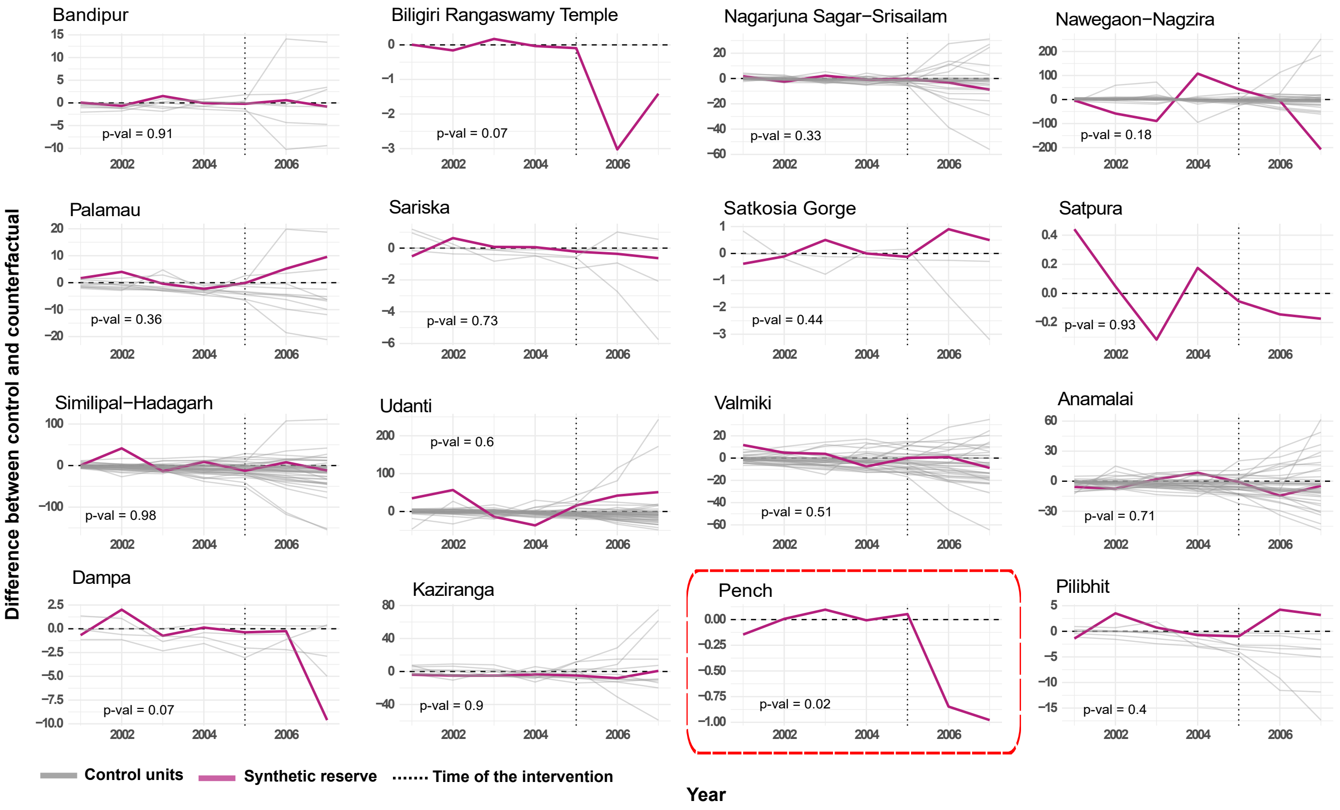

To test for anticipation effects, a backdated hypothetical intervention in 2005, which coincides with the constitution of the National Tiger Conservation Authority was implemented. Reserves that also exhibited significant effects from this pseudo-intervention were ruled out from the analyses due to the influence of potential anticipation effects. Of the 16 reserves from Supplementary Table 2 that exhibited significant effects of the enhanced conservation policy on deforestation rates (based on placebo testing using a two-sided Fisher's exact test to compare the ratios of pre-intervention and post-intervention mean squared prediction errors between placebo and treated units), only Pench Tiger Reserve exhibited significant anticipation effects (p-value = 0.02). The placebo tests for this reserve are enclosed in the dashed red box. To reject the null hypothesis, i.e. that there is no significant effect of the hypothetical intervention on deforestation, the mean squared prediction error ratios for the placebo units and the treated unit must be significantly different based on a two-sided Fisher's exact test. The vertical axis represents the difference between observed cumulative forest loss and counterfactuals for the reserves that demonstrated significant results (unadjusted p-value < 0.05). The solid pink line represents the synthetic control model while the light grey lines represent placebo tests Placebo cases with a pre-period mean squared prediction error (mspe) exceeding five times the treated unit pre-period mspe have been pruned. Significance levels from placebo testing have been reported for each synthetic counterfactual in the displayed plots.

Supplementary Tables

Supplementary Table 1: Complete list of reserves considered in this study with information on geographical grouping, year of establishment, tiger conservation policy intervention year, total forest area of reserve, total forest area in the baseline year (2000), and total forest loss by the end of 2020 in hectares. For untreated reserves, the intervention year is denoted by a '-' since these reserves did not undergo the conservation policy.

Protected area	*Year of establishment*	*Intervention year*	*Total area of reserve (ha)*	*Forest cover in 2000 (ha)*	*Total loss in 2001-2020 (ha)*
Shivalik-Gangetic , Central India, Sunderbans and Eastern Ghats					
Achanakmar	1975	2009	94,147.46	78,090.05	435.58
Amrabad	1978	2015	273,800.54	24,790.40	13.86
Badrama	1962	-	48,394.47	41,967.18	82.09
Bandhavgarh	1968	2007	48,175.18	20,315.54	10.06
Barnawapara	1976	-	24,990.65	11,855.35	53.78
Bhairamgarh	1983	-	1,094.07	1,072.80	31.90
Bhamragarh	1997	-	17,246.36	16,726.18	217.52
Bhoramdev	2001	-	35,599.05	24,663.22	96.00
Bor	1970	2012	18,609.61	717.45	0.00
Bori	1977	-	65,254.36	29,695.55	16.31
Chaprala	1986	-	11,697.51	1,298.42	21.69
Corbett	1936	2010	95,436.40	78,897.92	265.69
Dalma	1976	-	14,031.71	3,571.60	7.01
Debrigarh	1985	-	32,243.17	7,124.55	38.36
Dudhwa	1977	2010	126,219.92	96,180.23	53.93
Eturnagaram	1953	-	134,449.23	86,772.17	1,150.39
Guru Ghasidas	1981	-	104,550.00	51,259.62	16.56
Hadgarh	1978	-	12,543.46	2,195.01	30.74
Indravati	1982	2009	127,909.22	114,726.32	429.65
Jamwa Ramgarh	1982	-	23,064.50	26.06	0.00
Kanger Ghati	1982	-	20,096.60	17,915.42	109.17
Kanha	1955	2007	93,375.28	78,852.59	98.97
Karlapat	1992	-	6,290.98	5,944.20	295.71
Katepurna	1988	-	4,349.93	3.85	0.00
Kawal	1965	2012	69,359.40	11,874.67	184.92
Kinnersani	1977	-	60,018.55	38,425.49	2,802.87
Kothgarh	1981	-	43,228.08	31,854.41	8,927.91
Kuldiha	1984	-	25,097.42	10,341.22	106.25
Kuno	1981	-	39,512.03	226.97	0.00
Madhav	1959	-	34,726.62	200.12	29.96
Mahauadanr	1976	-	8,047.20	5,917.47	7.34
Mansinghdeo	2010	-	22,275.94	3,545.26	56.09
Melghat	1985	2007	192,848.51	43,251.33	10.78
Mukundra	1955	2013	28,374.77	9.18	4.59
Nagarjuna Sagar-Srisailam	1978	2007	333,930.29	110,547.36	67.89
Nandhaur	2012	-	25,587.59	24,684.56	148.44

Nawegaon-Nagzira	1975	2013	82,653.66	48,410.77	713.33
Noradehi	1984	-	135,961.73	7,874.22	88.85
Pachmarhi	1977	-	37,533.43	16,075.89	10.71
Painganga	1986	-	45,200.90	1,241.94	0.63
Palamau	1976	2012	97,297.55	66,990.56	119.38
Pamed Wild Buffalo	1985	-	45,170.21	30,318.85	155.41
Panna (Gangau)	1981	2007	60,157.23	7,440.18	2.97
Panpatha	1983	-	17,769.78	5,585.90	1.66
Papikonda	2008	-	96,092.11	90,333.28	230.74
Pench (Indira Priyadarsini)	1975	2007	74,208.42	29,453.78	15.84
Phen	1983	-	11,390.60	10,466.64	14.73
Pilibhit	2014	2014	59,595.84	52,683.85	389.36
Rajaji	1983	2015	79,436.18	60,837.89	53.64
Ranipur	1977	-	21,234.30	7,251.99	1.17
Ranthambhore	1980	2007	136,343.90	95.58	2.07
Ratapani	1978	-	104,056.46	13,936.70	6.33
Sanjay Dubri	1975	2011	72,848.37	34,797.65	15.08
Sariska	1955	2007	78,870.35	4,751.86	4.95
Satkosia Gorge	1976	2007	92,701.15	74,887.84	117.80
Satpura	1981	2007	34,989.82	22,014.80	6.84
Shoolpaneswar (Dhumkhal)	1982	-	65,392.71	4,221.18	35.01
Similipal-Hadagarh	1978	2007	236,178.76	204,781.16	672.26
Singhori	1976	-	24,764.47	4,089.43	7.37
Sohagibarwa	1987	-	24,399.62	20,767.60	50.15
Sohelwa	1988	-	62,027.43	53,822.14	165.78
Sri Penusila Narasimha	1997	-	108,223.32	16,595.88	42.06
Sunabeda	1988	-	66,408.02	25,207.13	123.05
Tadoba Andhari	1955	2007	79,392.72	21,043.92	11.13
Tamor Pingla	1978	-	73,694.38	34,402.48	8.28
Tipeshwar	1997	-	13,939.46	61.11	0.00
Udanti	1983	2009	192,183.51	111,588.31	1,856.05
Umred Karhandla	2012	-	17,838.32	3,683.15	59.29
Valmiki	1978	2012	78,436.25	73,532.48	274.42
Lothian Island	1976	-	3,494.50	173.88	0.00
Sundarbans	1984	2007	240,690.30	30,963.60	7.20
West Sunderban	2013	-	34,662.79	1,417.41	2.70
	North-East Hills and Brahmaputra				
Buxa	1986	2009	38,478.28	35,156.53	897.65
Dampa	1985	2007	38,529.41	38,503.53	375.12
Dibang	1991	-	465,079.39	179,024.94	817.81
Dibru-Saikhowa	1999	-	23,260.95	17,444.36	2,304.60
Dihing Patkai	2004	-	22,487.95	22,239.89	85.77
Eagle Nest	1989	-	21,799.00	21,398.19	27.05
East Karbi Anglong	2000	-	10,685.71	9,031.78	2,997.82
Fakim	1980	-	3,405.65	3,410.27	6.37
Garampani	1952	-	78.22	75.96	1.39

Gorumara	1992	-		7,098.66	6,598.03	35.03
Intangki	1993	-		26,912.01	26,171.63	1,352.57
Itanagar	1978	-		13,674.19	13,597.66	282.34
Jaldapara	1941	-		24,287.08	16,773.76	606.57
Kamlang	1989		2016	78,586.61	72,045.94	230.65
Kane	1991	-		3,171.47	3,141.79	35.99
Kaziranga	1974		2007	43,581.10	22,471.90	297.25
Mahananda	1976	-		16,571.71	14,340.92	61.00
Mahao	1980	-		27,984.10	25,595.46	413.28
Manas	1990		2008	186,173.86	152,879.56	3,722.45
Marat Longri	2003	-		30,340.85	28,338.53	6,002.94
Mouling	1986	-		48,591.83	48,501.13	35.34
Nambor	2000	-		1,662.64	1,660.83	3.94
Nambor-Doigrung	2003	-		4,938.27	4,701.42	66.10
Neora Valley	1986	-		17,487.65	16,280.38	58.77
Ngengpui	1991	-		5,291.75	5,251.41	46.57
North Karbi Anglong	2000	-		19,571.01	19,530.50	920.57
Orang	1999		2016	6,518.89	1,332.39	41.63
Pakke	1977		2012	82,191.21	80,866.63	662.03
Pangolakha	2002	-		12,099.70	7,031.87	51.69
Sessa Orchid	1989	-		6,546.55	6,136.18	20.96
Sonai Rupai	1998	-		20,235.70	15,295.44	4,814.88
Tale Valley	1995	-		23,868.80	23,767.27	48.51
Tawi	1978	-		3,515.79	3,385.59	412.13
Tokalo	2007	-		6,690.76	6,567.26	344.05
Yangoupokpi Lokchao	1989	-		16,646.71	14,892.72	2,453.85
Yordi-Rabe Supse	1996	-		38,581.18	36,105.81	54.66
	Western Ghats					
Anamalai	1976		2007	96,453.68	75,681.76	650.28
Anamudi Shola	2003	-		1,840.88	1,838.21	33.16
Aralam	1984	-		5,117.54	5,144.44	1.89
Bandipur	1974		2007	95,748.77	54,170.38	39.18
Bannerghatta	1974	-		20,272.49	1,776.15	2.38
Bhadra	1974		2007	46,320.53	41,959.02	108.95
Bhimgad	2010	-		15,689.77	11,907.99	13.05
Biligiri Rangaswamy Temple	2011		2011	55,224.37	40,064.66	43.50
Brahmagiri	1974	-		18,938.73	17,981.08	41.02
Cauvery North	2014	-		120,736.40	31,333.42	92.45
Cauvery	1987	-		110,741.68	18,145.85	58.47
Chimmony	1984	-		8,068.57	7,259.23	22.95
Chinnar	1984	-		10,804.56	8,186.73	72.91
Cotigaon	1968	-		10,043.65	9,717.48	30.19
Dandeli Anshi	1987		2007	137,822.83	120,681.84	188.20
Eravikulam	1978	-		11,679.71	6,011.37	108.74
Idukki	1976	-		14,273.49	7,736.06	110.46
Kalakad	1976		2007	85,737.57	65,416.95	527.76

Kannyakumari	2002	-		45,021.60	35,963.90	1,371.06
Kodaikanal	2013	-		101,896.23	79,068.37	2,793.18
Kottiyoor	2011	-		3,520.73	3,482.49	13.75
Kudremukh	1987	-		79,567.03	64,880.75	171.46
Kurinjimala	2006	-		987.31	908.27	73.77
Madei	1999	-		26,642.32	20,642.12	80.45
Malabar	2010	-		5,206.03	4,618.66	18.42
Malai Mahadeshwara	2013	-		81,140.38	34,587.17	24.45
Mathikettan Shola	2003	-		2,240.69	2,240.82	3.99
Megamalai	2009	-		36,836.81	18,816.58	100.01
Mollem	1992	-		24,588.05	22,148.46	34.87
Mookambika	1974	-		55,580.71	42,550.44	481.19
Mudumalai	1942		2007	33,199.49	26,291.50	21.17
Mukurthi	1990	-		9,109.32	4,019.22	310.10
Nagarahole	1988		2007	67,976.35	59,026.09	203.01
Nellai	2015	-		33,942.97	22,409.50	231.30
Netravali	1999	-		17,097.23	16,191.35	29.94
Neyyar	1958	-		10,409.02	9,733.61	148.37
Nugu	1974	-		3,158.62	86.53	0.89
Pambadum Shola	2003	-		1,305.59	1,275.34	48.50
Parambikulam	1973		2009	29,912.96	27,588.93	61.59
Peechi-Vazhani	1958	-		14,946.52	13,912.30	45.54
Peppara	1983	-		9,331.19	8,376.37	162.17
Periyar	1982		2007	58,149.10	51,667.38	229.18
Purna	1990	-		17,910.84	3,502.07	5.81
Pushpagiri	1987	-		11,939.91	10,907.39	81.59
Radhanagari	1958	-		41,338.54	18,131.40	57.73
Sahyadri	1985		2012	82,102.08	24,174.95	25.27
Sathyamangalam	2008		2013	78,851.21	35,414.63	38.08
Sharavati Valley	1974	-		45,846.33	26,275.66	273.87
Shendurney	1984	-		19,272.62	18,816.48	133.27
Shettihalli	1974	-		35,436.46	23,267.40	480.62
Silent Valley	1984	-		9,835.50	8,968.98	36.20
Someshwara	1974	-		38,607.00	37,561.13	126.52
Srivilliputhur Grizzled Squirrel	1988	-		41,517.00	18,669.44	163.65
Talakaveri	1987	-		11,731.96	10,835.65	10.18
Thattekad Bird	1983	-		1,372.94	1,320.17	1.44
Wayanad	1973	-		8,239.57	8,123.09	4.49

Supplementary Table 2: Synthetic control outputs for counterfactual Tiger Reserves. Reserves with significant results (unadjusted p-value < 0.05 based on placebo tests using a two-sided Fisher's exact test to compare the ratios of pre-intervention and post-intervention mean squared prediction errors between placebo and treated units) are highlighted in green. The ratio of the mean squared prediction error (mspe) of the counterfactual unit as compared with observed values is calculated before and after the intervention takes place. The mspe ratios for the placebo units and the treated unit must be significantly different to rule out whether the observed effect of the intervention on the outcome variable was a chance event. For each Tiger Reserve, the intervention year, mspe values, number of untreated donor reserves used in creating the synthetic control model, avoided forest loss estimates in ha, mean and uncertainty values for AGBC and BGBC in tCO2eq/ha and corresponding equivalent emissions in tCO2eq have been reported below. Pench (highlighted in orange) was eventually excluded due to anticipation effects (see Supplementary Figures 5, 6 and Supplementary Table 3).

Tiger Reserve	Intervention year	Mspe pre-treatment	Mspe post-treatment	Mspe ratio	Placebo test p-value	Number of donor units	Avoided forest loss (ha)	Mean AGBC in treatment year (tCO2eq/ha)	Mean AGBC uncertainty in treatment year (tCO2eq /ha)	Mean BGBC in treatment year (tCO2eq/ha)	Mean BGBC uncertainty in treatment year (tCO2eq /ha)	Mean equivalent emissions (tCO2eq)	Equivalent emissions uncertainty (tCO2eq)
Nawegaon-Nagzira	2013	9681.00	2930730.27	302.73	0.04	44	2645.31	35.54	17.96	10.58	9.68	416954.46	239672.09
Udanti	2009	848.39	599842.09	707.03	0.02	44	1610.52	37.35	14.91	11.00	8.88	285779.27	140678.37
Similipal-Hadagarh	2007	334.55	554451.42	1657.31	0.02	44	1569.87	52.94	9.90	15.77	11.74	395898.97	124658.71
Valmiki	2012	46.16	34919.60	756.45	0.04	44	314.81	84.82	34.08	23.33	17.49	119560.84	55507.00
Nagarjuna Sagar-Srisailam	2007	8.36	6216.61	743.49	0.04	44	166.61	22.83	9.67	8.39	6.92	19092.87	10140.78
Palamau	2012	16.88	7934.92	470.03	0.02	44	142.80	41.35	17.33	15.12	10.15	28011.95	13337.46
Satkosia Gorge	2007	0.18	981.06	5459.26	0.02	44	39.28	43.80	13.77	12.31	9.94	8087.46	3418.71
Bandipur	2007	0.59	174.47	294.15	0.04	44	24.89	24.22	10.22	7.90	7.08	2935.01	1580.54
Biligiri Rangaswamy Temple	2011	0.22	286.87	1306.83	0.02	44	21.79	36.44	8.60	10.71	9.18	3684.42	1348.43
Sariska	2007	0.15	66.56	455.14	0.02	44	13.64	6.43	4.65	7.09	4.26	676.89	446.05
Satpura	2007	0.05	36.32	728.79	0.02	44	8.68	41.45	13.15	13.49	10.55	1750.04	755.02
Pench	2007	0.01	13.64	1059.64	0.02	44	-7.61	39.88	19.09	11.87	10.32	-1445.63	-821.84
Kaziranga	2007	4.66	3079.99	661.17	0.03	29	-56.92	35.79	3.77	10.46	9.99	-9661.57	-2875.12
Dampa	2007	1.88	5077.83	2700.65	0.03	29	-191.05	75.88	16.16	19.55	15.56	-66907.37	-22246.51
Anamalai	2007	31.96	33263.49	1040.70	0.02	44	-207.77	35.25	9.18	10.41	9.05	-34818.51	-13896.06
Pilibhit	2014	30.39	18740.19	616.61	0.02	44	-300.04	74.32	30.50	19.82	18.90	-94928.02	-46045.64
Periyar	2007	160.27	20046.53	125.08	0.07	44	304.13	47.40	16.49	12.44	10.96	66786.24	30653.11
Manas	2008	5077.01	778210.61	153.28	0.07	29	-1686.52	47.25	22.53	13.62	12.62	-376743.72	-218075.85
Achanakmar	2009	536.53	136312.69	254.06	0.07	44	733.41	46.16	22.76	13.89	12.20	161620.41	94111.86
Indravati	2009	263.14	124639.96	473.67	0.07	44	694.87	44.79	23.50	12.53	12.83	146180.59	92646.50
Sahyadri	2012	2.47	598.14	242.52	0.09	44	40.46	37.06	10.87	10.30	8.77	6726.72	2655.09
Nagarahole	2007	21.15	1212.25	57.33	0.11	44	-1.93	32.29	11.66	9.88	9.62	-298.39	-150.61
Panna	2007	0.02	4.67	221.03	0.11	44	2.75	13.66	7.02	7.78	5.09	216.41	122.23
Kanha	2007	8.19	1420.87	173.54	0.13	44	74.54	52.63	24.29	16.14	14.43	18814.91	10594.18
Parambikulam	2009	8.30	430.23	51.81	0.13	44	-24.23	45.99	12.86	12.20	11.68	-5174.26	-2182.29

Kawal	2012	164.98	1896.66	11.50	0.13	44	54.68	28.09	13.83	8.72	8.34	7036.97	4103.24
Dandeli Anshi	2007	2.40	335.95	140.04	0.16	44	1.48	44.64	13.50	12.18	10.08	308.15	127.94
Sundarbans	2007	0.01	3.09	289.15	0.16	44	-1.35	19.63	8.50	6.55	5.19	-129.38	-67.67
Sanjay Dubri	2011	0.53	24.01	45.71	0.18	44	5.80	37.11	17.56	13.70	10.45	1052.78	574.37
Amrabad	2015	1.04	31.49	30.42	0.20	44	8.34	10.18	5.18	5.79	4.37	400.09	225.29
Mudumalai	2007	0.13	4.36	33.73	0.29	44	-3.96	29.11	8.34	8.99	8.28	-553.27	-241.31
Dudhwa	2010	1.31	29.67	22.68	0.29	44	6.87	59.75	25.70	16.92	14.51	1933.76	1014.05
Kalakad	2007	475.62	8219.60	17.28	0.38	44	188.06	37.85	11.32	10.86	9.22	33615.38	14179.07
Ranthambhore	2007	0.05	3.03	66.85	0.38	44	2.07	1.36	0.63	2.99	1.43	32.93	15.59
Pakke	2012	1639.94	13368.89	8.15	0.40	29	-167.23	77.51	33.69	19.06	15.81	-56928.11	-28409.64
Tadoba Andhari	2007	0.64	29.56	46.47	0.49	44	7.27	24.50	12.77	8.14	7.35	870.64	536.72
Bandhavgarh	2007	0.79	37.45	47.63	0.51	44	8.11	33.57	15.20	13.35	9.32	1397.08	730.17
Bor	2012	0.20	1.32	6.61	0.53	44	1.24	3.46	3.41	2.71	2.14	25.56	23.24
Sathyamangalam	2013	0.39	3.51	8.94	0.60	44	1.98	21.39	6.52	6.97	6.26	190.87	79.20
Bhadra	2007	9.35	89.31	9.55	0.69	44	25.16	43.34	10.62	11.89	11.80	5099.22	2070.05
Melghat	2007	0.32	5.67	17.73	0.69	44	-2.91	24.80	10.94	8.47	7.71	-354.69	-198.78
Buxa	2009	782.24	4855.75	6.21	0.73	29	120.63	56.43	25.16	15.20	13.78	31712.16	17302.23
Rajaji	2015	8.23	1.69	0.21	0.93	44	1.95	52.89	22.49	16.58	13.68	437.05	209.44
Corbett	2010	557.47	911.70	1.64	0.96	44	-17.11	62.07	28.90	19.07	14.97	-5094.93	-2754.36
Mukundra	2013	0.39	0.01	0.04	1.00	44	-0.04	1.45	0.62	2.73	1.29	-0.47	-0.18

Supplementary Table 3: Assessment of anticipation effects. Synthetic control outputs for a backdated intervention in 2005 for the 16 modelled Tiger Reserves from Supplementary Table 2 between the years 2001 and the intervention date. Reserves with significant results where unadjusted p-value < 0.05 (based on placebo tests using a two-sided Fisher's exact test to compare the ratios of pre-intervention and post-intervention mean squared prediction errors between placebo and treated units) are highlighted in orange and represent potential anticipation effects. Therefore, these reserves were excluded from the final results. The ratio of the mean squared prediction error (mspe) of the counterfactual unit as compared with observed values is calculated before and after the intervention takes place. The mspe ratios for the placebo units and the treated unit must be significantly different to rule out whether the observed effect of the intervention on the outcome variable was a chance event. For each Tiger Reserve, the intervention year, mspe values, the number of untreated donor reserves used in creating the synthetic control model and avoided forest loss estimates in ha have been reported below. Pench (highlighted in orange) was eventually excluded due to anticipation effects (see Supplementary Figures 5 and 6).

Tiger Reserve	Mspe pre-treatment	Mspe post-treatment	Mspe ratio	Placebo test p-value	Number of donor units	Anticipation effect (ha)
Anamalai	32.70	115.19	3.52	0.71	44	4.67
Bandipur	0.55	0.53	0.96	0.91	44	0.83
Biligiri Rangaswamy Temple	0.01	3.34	257.69	0.07	44	2.63
Dampa	1.04	46.18	44.39	0.07	29	9.61
Kaziranga	20.51	33.26	1.62	0.90	29	-0.74
Nagarjuna Sagar-Srisailam	3.24	44.45	13.74	0.33	44	8.86
Nawegaon-Nagzira	4985.36	939743.04	188.50	0.18	44	1593.14
Palamau	4.87	227.48	46.68	0.36	44	-12.63
Pench	0.01	0.84	124.96	0.02	44	0.98
Pilibhit	3.28	132.80	40.47	0.40	44	-23.00
Sariska	0.14	0.27	1.83	0.73	44	0.63
Satkosia Gorge	0.09	0.53	6.22	0.44	44	-0.50
Satpura	0.07	0.03	0.38	0.93	44	0.17
Similipal-Hadagarh	429.22	102.83	0.24	0.98	44	11.97
Udanti	1243.04	7798.13	6.27	0.60	44	-116.12
Valmiki	46.70	447.39	9.58	0.51	44	-39.79

Supplementary Table 4: Robustness checks using area-based trimming of donor pools. We used two thresholds where we first ensured that donor reserves must be at least a tenth of the size of a Tiger Reserve to be included in the donor pool for modelling the synthetic counterfactual. Similarly, we used a more conservative threshold, where a donor reserve must be at least a quarter of the size of a Tiger Reserve to be included in the analyses. We evaluated the direction, significance and magnitude of our modelled reserves with these adjusted donor pools to check for the robustness of our final results. Since at least 20 donor units are required to obtain significance values of less than 0.05, we could produce counterfactuals for 8 of the 15 tiger reserves described in our main results for the 25% threshold. However, our results were robust to trimmed donor areas with all trimmed outputs for both thresholds demonstrating the same direction (whether the effect was consistent in terms of avoided loss or increased loss). Additionally, 73% (11 out of 14) and 75% (6 out of 8) of the results with a trimmed donor pool of 10% and 25% respectively, were within our +-20% final findings. For each of the thresholds, the significance value from placebo testing, the number of reserves in the donor pool and avoided deforestation are included. Two additional columns indicate whether the results are within 20% of the final value and whether the direction of the effect is the same as the untrimmed result.

	**Untrimmed**				**10% threshold**					**25% threshold**				
**Tiger Reserve**	**Treatment year**	**Placebo test p-value**	**Number of donor units**	**Avoided forest loss (ha)**	**Placebo test p-value**	**Number of donor units**	**Avoided forest loss (ha)**	**Within 20%?**	**Direction**	**Placebo test p-value**	**Number of donor units**	**Avoided forest loss (ha)**	**Within 20%?**	**Direction**
Anamalai	2007	0.02	44	-207.77	0.03	31	-194.24	✓	✓	-	-	-	-	-
Bandipur	2007	0.04	44	24.89	0.06	31	31.64	x	✓	-	-	-	-	-
Biligiri Rangaswamy Temple	2011	0.02	44	21.79	0.03	35	27.37	x	✓	0.12	24	6.75	x	✓
Dampa	2007	0.03	29	-191.05	0.04	24	-162.06	✓	✓	-	-	-	-	-
Kaziranga	2007	0.03	29	-56.92	0.04	24	-86.75	x	✓	-	-	-	-	-
Nagarjuna Sagar-Srisailam	2007	0.04	44	166.61	0.05	21	168.22	✓	✓	-	-	-	-	-
Nawegaon-Nagzira	2013	0.04	44	2645.31	0.03	39	2327.36	✓	✓	0.03	30	2220.57	✓	✓
Palamau	2012	0.02	44	142.80	0.03	39	150.45	✓	✓	0.04	27	166.50	✓	✓
Pilibhit	2014	0.02	44	-300.04	0.02	41	-337.81	✓	✓	0.03	34	-340.66	✓	✓
Sariska	2007	0.02	44	13.64	0.37	40	4.78	x	✓	0.50	31	2.80	x	✓
Satkosia Gorge	2007	0.02	44	39.28	0.03	39	45.12	✓	✓	0.04	27	31.96	✓	✓
Satpura	2007	0.02	44	8.68	0.02	42	8.68	✓	✓	0.10	39	8.68	✓	✓
Similipal-Hadagarh	2007	0.02	44	1569.87	0.04	27	1569.88	✓	✓	-	-	-	-	-
Udanti	2009	0.02	44	1610.52	0.03	31	1596.74	✓	✓	-	-	-	-	-
Valmiki	2012	0.04	44	314.81	0.05	40	300.03	✓	✓	0.03	31	319.03	✓	✓
