# Supplementary material for: Climate co-benefits of tiger conservation
Source: Nat Ecol Evol. 2023 May 25;7(7):1104–13. doi: 10.1038/s41559-023-02069-x (PMC10333118; doi:10.1038/s41559-023-02069-x)
Supplement: Supplementary file 2 — Reporting Summary [file 41559_2023_2069_MOESM2_ESM.pdf]

nature portfolio

Corresponding author(s): Aakash Lamba, Lian Pin Koh

Last updated by author(s): Mar 11, 2023

Reporting Summary

Nature Portfolio wishes to improve the reproducibility of the work that we publish. This form provides structure for consistency and transparency in reporting. For further information on Nature Portfolio policies, see our Editorial Policies and the Editorial Policy Checklist.

Statistics

For all statistical analyses, confirm that the following items are present in the figure legend, table legend, main text, or Methods section.

n/a	Confirmed	
☐	☒	The exact sample size (n) for each experimental group/condition, given as a discrete number and unit of measurement
☒	☐	A statement on whether measurements were taken from distinct samples or whether the same sample was measured repeatedly
☐	☒	The statistical test(s) used AND whether they are one- or two-sided<br>*Only common tests should be described solely by name; describe more complex techniques in the Methods section.*
☐	☒	A description of all covariates tested
☒	☐	A description of any assumptions or corrections, such as tests of normality and adjustment for multiple comparisons
☐	☒	A full description of the statistical parameters including central tendency (e.g. means) or other basic estimates (e.g. regression coefficient) AND variation (e.g. standard deviation) or associated estimates of uncertainty (e.g. confidence intervals)
☐	☒	For null hypothesis testing, the test statistic (e.g. F, t, r) with confidence intervals, effect sizes, degrees of freedom and P value noted<br>*Give P values as exact values whenever suitable.*
☒	☐	For Bayesian analysis, information on the choice of priors and Markov chain Monte Carlo settings
☒	☐	For hierarchical and complex designs, identification of the appropriate level for tests and full reporting of outcomes
☐	☒	Estimates of effect sizes (e.g. Cohen's d, Pearson's r), indicating how they were calculated

Our web collection on statistics for biologists contains articles on many of the points above.

Software and code

Policy information about availability of computer code

Data collection	All data used in the analyses were based on published studies or publicly available datasets, which have been listed in the methods section. Cumulative forest loss and spatial covariates were collected using Google Earth Engine (code included). QGIS ver. 3.22 was used to extract road length per reserve.
Data analysis	All data analyses were conducted in R version 4.2.0. Synthetic control models were generated using the 'tidysynth' package (ver. 0.1.0) in R.

For manuscripts utilizing custom algorithms or software that are central to the research but not yet described in published literature, software must be made available to editors and reviewers. We strongly encourage code deposition in a community repository (e.g. GitHub). See the Nature Portfolio guidelines for submitting code & software for further information.

Data

Policy information about availability of data

All manuscripts must include a data availability statement. This statement should provide the following information, where applicable:

- Accession codes, unique identifiers, or web links for publicly available datasets
- A description of any restrictions on data availability
- For clinical datasets or third party data, please ensure that the statement adheres to our policy

All data generated generated from the study can be found at https://doi.org/10.5281/zenodo.7711520

Human research participants

Policy information about studies involving human research participants and Sex and Gender in Research.

Reporting on sex and gender	na
Population characteristics	na
Recruitment	na
Ethics oversight	na

Note that full information on the approval of the study protocol must also be provided in the manuscript.

Field-specific reporting

Please select the one below that is the best fit for your research. If you are not sure, read the appropriate sections before making your selection.

☐ Life sciences ☐ Behavioural & social sciences ☒ Ecological, evolutionary & environmental sciences

For a reference copy of the document with all sections, see nature.com/documents/nr-reporting-summary-flat.pdf

Ecological, evolutionary & environmental sciences study design

All studies must disclose on these points even when the disclosure is negative.

Study description	We used a synthetic controls approach to model the effects of an enhanced tiger conservation policy intervention on reducing deforestation rates in protected areas in India. We split protected areas that fall within the IUCN tiger range map into a treatment group which underwent the conservation policy (Tiger Reserves) and a 'donor pool' of untreated protected areas that did not undergo the conservation intervention. We used a weighted model of the donor pool reserves to create a synthetic counterfactual that simulated forest loss rates in treated Tiger Reserves before the policy intervention took place. These synthetic models were extrapolated into the post-intervention period, where the differences between observed cumulative forest loss in Tiger Reserves and their corresponding synthetic counterfactuals were used to estimate avoided forest loss. We translated this avoided forest loss into a carbon emissions equivalent value based on the mean above-ground and below-ground carbon biomass density in these reserves. Finally, we estimated the ecosystem services values from the avoided social cost of carbon emissions and potential carbon offset revenues from avoided deforestation due to the enhanced protection of Tiger Reserves in India.
Research sample	Our research sample consisted of protected areas in mainland India that fall within the global tiger range map. For each of these reserves, the outcome variable i.e. cumulative forest loss and associated spatial co-variates were collected for each year between 2001 and 2020. The observed forest loss values were obtained from the Hansen Global Forest Change v1.9 dataset.
Sampling strategy	Protected areas were split into those that (1) underwent an enhanced tiger conservation policy and (2) protected areas that did not undergo this policy and were thus used to create a counterfactual model to simulate cumulative forest loss in (1) had the intervention not taken place.
Data collection	Our analyses relied on using publicly available spatial datasets which were collected and processed using Google Earth Engine and QGIS ver. 3.22. The full list of datasets used has been described in the methods section.
Timing and spatial scale	Our study includes data between 2000 and 2020, with 2001 representing the first year of forest loss. The response variable i.e. cumulative forest loss and associated spatial co-variates were reported annually per reserve between 2001 and 2020. Data were included for all protected areas in mainland India that fall within the IUCN tiger range map.
Data exclusions	We excluded protected areas that underwent the conservation intervention less than five years after the starting period of the study in 2020 and less than five years before the end of our study period in 2020. This ensured sufficient data in the pre-intervention period to allow robust model fitting and sufficient time in the post-intervention period to observe the effects of enhanced protection.
Reproducibility	We have provided the data and code used in our study to ensure reproducibility of our results. We ran our counterfactual simulations multiple times to ensure that results were repeatable. Additionally, we ran a series of robustness checks to validate our results, which included tests for anticipation effects and examining the robustness of the results to trimming control units in the donor pool.
Randomization	Randomization was not relevant to our study since we rely on comparing observational data with synthetically produced counterfactuals to produce our results.
Blinding	Blinding was not relevant to the study since it used spatial analyses that worked with historical data.

Did the study involve field work? ☐ Yes ☒ No

Reporting for specific materials, systems and methods

We require information from authors about some types of materials, experimental systems and methods used in many studies. Here, indicate whether each material, system or method listed is relevant to your study. If you are not sure if a list item applies to your research, read the appropriate section before selecting a response.

Materials & experimental systems

n/a	Involved in the study
☒	☐ Antibodies
☒	☐ Eukaryotic cell lines
☒	☐ Palaeontology and archaeology
☒	☐ Animals and other organisms
☒	☐ Clinical data
☒	☐ Dual use research of concern

Methods

n/a	Involved in the study
☒	☐ ChIP-seq
☒	☐ Flow cytometry
☒	☐ MRI-based neuroimaging
